# Case report: Toxic epidermal necrolysis as a unique presentation of acute graft versus host disease in a pediatric patient

**DOI:** 10.3389/fimmu.2024.1452245

**Published:** 2025-01-23

**Authors:** Elizabeth Marlowe, Rachel Palmer, April L. Rahrig, Devin Dinora, Jessica Harrison, Jodi Skiles, Mahvish Q. Rahim

**Affiliations:** ^1^ Pediatric Hematology Oncology and Stem Cell Transplant, Riley Hospital for Children at Indiana University (IU) Health, Indiana University School of Medicine, Indianapolis, IN, United States; ^2^ Indiana University School of Medicine, Indianapolis, IN, United States

**Keywords:** toxic epidermal necrolysis, graft versus host disease, pediatric, stem cell transplant, Stevens-Johnson syndrome

## Abstract

**Introduction:**

Acute graft versus host disease (aGVHD) is a common complication of stem cell transplant (SCT), with skin involvement being most common. Severe presentations of skin aGVHD involving rapid progression of rash to bullae formation and mucosal involvement are rare. There are reports of patients with skin aGVHD that present with clinical characteristics mimicking toxic epidermal necrolysis (TEN), suggesting a possible overlap between the two. Management and outcomes of pediatric patients with this overlapping, severe presentation have rarely been described.

**Case presentation:**

This report describes an 11-year-old boy with refractory T-cell acute lymphoblastic leukemia who received peripheral blood SCT from a matched unrelated donor. Day 26 post-SCT, he developed a maculopapular facial rash, which progressed to the development of vesicles coalescing into bullae involving his conjunctiva, face, oral mucosa, and genital mucosa. Initially, systemic steroid monotherapy was initiated, but with rapid rash progression and mucosal involvement, intravenous immunoglobulin (IVIg) 2 g/kg divided over 5 days was added as management for suspected TEN-like aGVHD based on clinical findings. Ruxolitinib was subsequently started as adjunctive management for aGVHD. His skin findings continued to improve with near total resolution by day 49 post-SCT.

**Conclusion:**

We report a unique case of TEN-like aGVHD with rapid progression to >30% body surface area involvement including bullae formation and detachment of epidermis. There have been few case reports of similar presentations, most with poor outcomes. We aim to supplement the literature available by reporting our successful management with steroids, IVIg, and ruxolitinib, which resulted in early resolution of symptoms in a pediatric patient.

## 1 Introduction

Allogenic hematopoietic stem cell transplant (SCT) offers a curative therapy option for patients with underlying hematological malignancies. Allogenic SCT can result in improved disease-free survival from graft-versus-malignancy effect against the patient’s primary malignancy (1, 2). The benefit of graft-versus-malignancy is accompanied by the risk for graft-versus-host disease (GVHD), an often-formidable complication of SCT.

GVHD occurs when antigens of the recipient are expressed to donor T cells and result in donor T-cell activation and immune response to the host, resulting in tissue damage and inflammatory cytokine secretion (2–4). Risk factors for GVHD include human leukocyte antigen (HLA) mismatch, sex disparity between donor and recipient, the conditioning regimen, older age, multiparous female donors, and the source of the graft (3, 5). The development of GVHD can also present the potential for graft-versus-tumor (GVT) effect, which offers an antitumor effect against hematologic malignancies. Despite the possible benefits of GVT effects, careful balance between GVT desired effects and GVHD detrimental effects is imperative. GVHD prophylaxis with calcineurin inhibitors or mTOR inhibitors, in combination with methotrexate or mycophenolate mofetil, is employed to aide in minimizing risk of GVHD in non-malignant disease states and to prevent severe GVHD in malignant disease states where some GVT effect is desired (6).

GVHD is classified as acute (aGVHD) if occurring within the first 100 days post-SCT and chronic (cGVHD) if occurring beyond 100 days post-SCT. Additionally, persistent, recurrent, or late-onset acute GVHD can occur beyond 100 days post-SCT, and more recently, overlap syndrome GVHD has been elucidated as GVHD that has no time limit for presenting symptoms and is characterized by symptoms with features of both acute and chronic GVHD (7). GVHD biomarkers can be used to help predict the risk of developing severe aGVHD and non-relapse mortality. These biomarkers include regenerating islet-derived 3-α (Reg3α) and suppression of tumorgenicity 2 (ST2) (8). The skin is the most common site and usually the first organ involved with aGVHD, with onset occurring around the time of engraftment of donor cells, typically presenting with a maculopapular rash (9, 10). Gastrointestinal (GI) and liver involvement in aGHVD are less common, but can be characterized by excessive diarrhea and transaminitis (9). Acute GVHD is assigned an overall grade of severity through combinations of staging for skin, liver, and GI involvement (**Table 1**) via the Mount Sinai Acute GvHD International Consortium (MAGIC) criteria (11).

**Table 1:** MAGIC criteria for grading and staging of aGVHD (11).

Pediatric acute GVHD Staging		
Organ	Stage	Description
Skin	0	No rash
	1	Rash <25% of BSA
	2	Rash 25%–50% of BSA
	3	Rash >50% of BSA
	4	Generalized erythroderma (>50% BSA) plus bullous formation and desquamation >5% of BSA
Liver	0	Total serum bilirubin <2 mg/dL
	1	Total serum bilirubin 2–3 mg/dL
	2	Total serum bilirubin 3.1–6 mg/dL
	3	Total serum bilirubin 6.1–15 mg/dL
	4	Total serum bilirubin >15 mg/dL
Lower GI	0	Diarrhea <10 mL/kg/day or <4 episodes/day
	1	Diarrhea 10–19.9 mL/kg/day or 4–6 episodes/day
	2	Diarrhea 20–30 mL/kg/day or 7–10 episodes/day
	3	Diarrhea >30 mL/kg/day or >10 episodes/day
	4	Severe abdominal pain with or without ileus or grossly blood stools (regardless of stool volume)
Upper GI	0	No or intermittent anorexia or nausea or vomiting
	1	persistent anorexia or nausea or vomiting
Pediatric acute GVHD Grading Systems		
Consensus grading		
0	No organ involvement stage 1–4	
I	Stage 1–2 skin WITHOUT liver, upper GI or lower GI involvement	
II	Stage 3 rash and/or stage 1 liver and/or stage 1 upper GI and/or stage 1 lower GI	
III	Stage 2–3 liver and/or stage 2–3 lower GI WITH stage 0–3 skin and/or stage 0–1 upper GI	
IV	Stage 4 skin, liver, OR lower GI involvement WITH stage 0–1 upper GI	

*Each grade is based on maximum stage for each involved organ.

GVHD is commonly treated through suppression of the immune system with the use of topical and/or systemic steroids based on the severity of aGVHD. Transplant-related mortality is high in patients who do not respond to steroids within the first 5 days of treatment (6).

Stevens–Johnson Syndrome (SJS) and toxic epidermal necrolysis (TEN) are severe cutaneous adverse reactions that often occur as medication-related adverse reactions. TEN is on a spectrum with SJS, in which SJS is defined as <10% of body surface area (BSA) and TEN involves >30% BSA with blisters evolving to bullae, and ultimately leading to erosions or sheets of skin detachment that exposes erythematous dermis tissue. Overlapping SJS/TEN occurs between 10% and 30% BSA (12). SJS/TEN is a life-threatening rapid progression of blisters and lesions involving both the skin and mucosal membranes (eyes, mouth, and genitalia). The pathophysiology behind SJS/TEN is related to keratinocyte apoptosis with epidermal necrosis and dermo-epidermal separation (12). This process is often cytokine driven and studies have associated elevated levels of interferon gamma (IFN-γ), IL-6, IL-8, IL-15, TNF- α, and others with its occurrence (12, 13).

Management of SJS/TEN often includes supportive care in a burn unit, withdrawal of the inciting agent, and in some situations, early systemic corticosteroids (12, 14, 15). Studies have looked at the utility of using IVIg as treatment for SJS/TEN but have found inconsistent results demonstrating benefit (16–18). Two studies found that IVIg in combination with systemic steroids have resulted in a significantly lower mortality compared to treatment with corticosteroids alone and that the combination also potentially reduces a patient’s recovery time (19, 20).

SJS/TEN is not typically described as a stem cell transplant-related complication. However, there have been several case reports of adult patients post-transplant who develop a severe form of aGVHD of the skin, which can be confounded with SJS/TEN characteristics (21–24). There is a paucity of pediatric data regarding this overlap of aGVHD and SJS/TEN characteristics. Although SJS/TEN and aGVHD can clinically present similarly and be difficult to distinguish, their treatments differ and pose a diagnostic dilemma. While typical management of aGVHD includes utilization of immunosuppression with steroids or Ruxolitinib, SJS/TEN treatment can include steroids, cyclosporine, etanercept, infliximab, and even IVIg (12). Better understanding of these two diagnoses will aid in early recognition and appropriate therapeutic interventions. Here, we describe the case of an 11-year-old boy presenting with aGVHD with TEN features, including diagnostic and management approaches.

## 2 Case description

We present an 11-year-old boy with refractory T-cell acute lymphoblastic leukemia (T-ALL), who received a peripheral blood SCT from a 12/12 HLA-matched unrelated donor. Preparative regimen included total body irradiation (12 Gy) with testicular boost and cyclophosphamide (60 mg/kg/dose, IV, D-3 and D-2). His GVHD prophylaxis regimen included methotrexate (15 mg/m^2^ on D+1, then 10 mg/m^2^ on D+3, D+6, and D+11) and cyclosporine (goal, 200–250). He achieved engraftment with 100% donor whole blood and T-cell chimerism 21 days (D+21) post-SCT.

**Figure 1:**
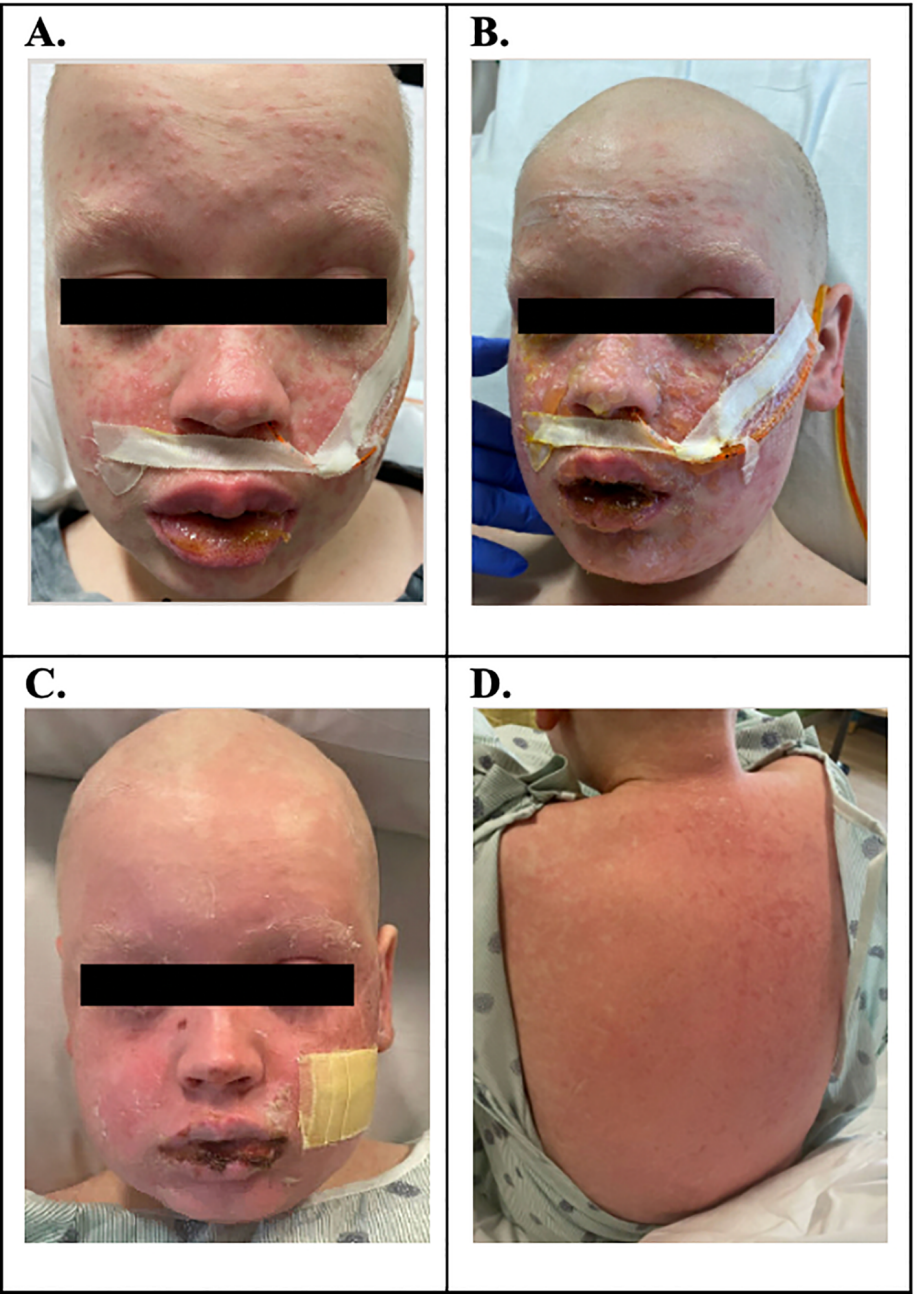
**(A)** D+26: maculopapular facial rash. **(B)** D+27: progression of rash over 80% BSA; blistering rash with involvement of mouth and genital mucosa; injected sclera with blurry vision; skin biopsy confirmed pathologic Grade III aGVHD. **(C, D)** D+34: bullae slowly ruptured, skin peeled, and the patient was left with erythematous skin to the face, chest, back, and extremities.

On D+23, he developed lip swelling of unknown etiology that was unresponsive to diphenhydramine (0.5 mg/kg, NG, q6h PRN). Three days later (D+26), he was found to have a maculopapular facial rash, which rapidly progressed over 24 h to involve approximately 80% of his total BSA with skin detachment >30% BSA (**Figure 1A**). The progression was notable for development of vesicles coalescing into bullae and for mucosal involvement of his mouth, eyes, scrotum, and urethra (**Figure 1B**). Ocular involvement included injected sclera and blurry vision with ophthalmology exam noting 1+ conjunctival injection and 2+ blepharitis bilaterally. Dermatology work-up for pemphigus IgG was negative, and broad infectious work-up was performed, which resulted as follows: HSV swab of vesicular lesion and serum PCR negative, serum HHV6 PCR negative, mycoplasma IgG elevated, and normal mycoplasma IgM results. Adverse reaction to medications was considered as well; however, no new medications within 23 days of the onset of rash (cyclosporine was started 29 days prior to the onset of rash, and cefepime was started 23 days prior). To note, he was not on trimethoprim-sulfamethoxazole, which is notorious for causing SJS/TEN, and while commonly used in oncology patients as prophylaxis for pneumocystis jerovecii pneumonia, this is typically avoided prior to engraftment post-SCT due to the adverse effect of bone marrow suppression. He received empiric azithromycin (500 mg, IV, q24h) for 5 days for possible mycoplasma pneumoniae-induced rash and mucositis while lab testing was pending. A skin biopsy of his right upper back confirmed pathological Grade III skin aGVHD, using the MAGIC Criteria. With confirmation of aGVHD on D+27, he was started on systemic steroids (methylprednisolone IV 2 mg/kg/day) in addition to topical steroids (desonide 0.05% topical cream BID and triamcinolone 0.1% topical cream TID). Due to ocular involvement, neomycin/polymyxin B/dexamethasone eye drops were also initiated. An aGVHD biomarker algorithm panel, including Reg3α and ST2, was sent on D+7, D+27, and D+34, and all resulted showing values indicative of low risk for severe aGVHD.

Despite the initiation of steroids, the rash continued to progress rapidly over the next 24 h with bullae formation, worsening mucosal involvement, and desquamation of the skin. He progressed to clinical Grade IV stage IV aGVHD of the skin (see **Table 1**). He continued to complain of blurry vision and eye discomfort warranting the addition of cyclosporine 0.05% eye drops. Given the rapid progression, presentation severity, and mucosal involvement, there was concern for an unusual presentation of aGVHD, possibly on the spectrum of TEN. With the concern for TEN-like aGVHD, IVIg 2 g/kg divided over 5 days was started as an adjunctive management on D+28. IVIg was tolerated well without allergic reaction or symptoms of aseptic meningitis. With the addition of IVIg, the patient’s rash stabilized after one dose without further progression. In the 4 days following IVIg initiation, the presence of the rash persisted. While not worsening, given persistence of the rash without overwhelming improvement, ruxolitinib 5 mg orally twice daily was added on D+32. Over the next 2 days, the bullae slowly ruptured, the skin peeled, and the patient was left with erythematous skin on D+34 (**Figures 1C, D**). His skin findings continued to improve with near total resolution by D+49 (**Table 2**).

**Table 2:** Clinical Timeline.

Clinical Timeline	Days s/p SCT	Intervention Timeline
WBC engraftment (100% donor chimerism)	**D+21**	
Lip swelling of unknown etiology	**D+23**	Diphenhydramine with no response
Maculopapular facial rash (**Figure 1A**)	**D+26**	
Progression of rash to 80% total body surface area; blistering rash to mouth and genital mucosa; injected sclera with blurry vision; skin biopsy confirmed pathologic Grade III aGVHD (**Figure 1B**)	**D+27**	Started topical steroids (triamcinolone, desonide) and systemic steroids (methylprednisolone); started neomycin/polymyxin B/dexamethasone eyedrops
Increasing bullae with skin peeling and worsening of mucosal involvement, despite systemic steroids	**D+28**	IVIg 2 g/kg divided over 5 days for TEN treatment
Stabilization of skin rash; worsening bilateral bulbar conjunctival injection	**D+29**	Started cyclosporine eyedrops
Rash remained stable; concern for continued underlying aggressive steroid-refractory aGVHD	**D+32**	Started ruxolitinib
Continued corneal irritation and blurry vision	**D+37**	Amniotic membrane lenses placed in eyes bilaterally
Near total resolution of rash	**D+49**	

The bold print represent the characteristics of the patient we are presenting.

He continued to have ocular symptoms despite topical therapy; so on D+37, he had amniotic membrane lenses placed bilaterally for corneal protection in addition to continuation of cyclosporine eye drops. The amniotic membrane lenses placed by ophthalmology were removed on D+55.

As the patient continued to recover in the ensuing months, medications were further tapered and eventually discontinued. On D+77, ruxolitinib was stopped, and approximately 6 months post-SCT, the cyclosporine eyedrops were stopped. The patient required a prolonged steroid course with prednisone taper schedule of 10% weekly, followed by transition to hydrocortisone taper when down to adrenal dose steroids, with complete steroid discontinuation approximately 6 months post-SCT. The patient’s most recent bone marrow evaluation at 1-year post-transplant was negative for leukemia. His aGVHD remains quiescent with no concerns for chronic GVHD. The patient is now 24 months from SCT, in remission, back in school and is doing very well.

## 3 Discussion

Ultimately, this patient’s presentation was most consistent with toxic epidermal necrolysis as a presentation of aGVHD, given the rapid onset with facial and mucus membrane involvement with associated detachment. Severe aGVHD (stage 4) of the skin occurs in only 11% of pediatric SCT patients and can closely mimic TEN with severity and extent of skin involvement (25). As our patient presentation highlights, it is vital to distinguish between these two entities as the ultimate treatment approach is dependent on the underlying diagnosis. There are multiple measures that can be used to distinguish these entities (see **Table 3**) (26). Temporally, our patient’s presentation was consistent with aGVHD, as his symptoms developed around the time of WBC engraftment. TEN typically presents within 4–6 weeks of starting a new medication. In our patient’s case, his symptoms started ~3.5 weeks from his preparative chemotherapy, and there were no new medications or changes that would lead to identification of a specific medication related trigger. If new medications are being considered as a trigger for SJS/TEN, the ALDEN algorithm can be considered to assess for drug causality. In our patient, there was no new initial drug for us to score for drug causality, so we did not use this algorithm. Alternatively, causation for TEN presentation of aGVHD in our patient may have been the receipt of donor T cells.

**Table 3:** Comparison of timing, clinical presentation, and treatment strategies in TEN and aGVHD of the Skin (26, 29).

	TEN	aGVHD
**Timing**	Within 4–6 weeks of new medication	**<100 days post-SCT**, typically with WBC engraftment With taper or withdrawal of immune suppression
**Clinical presentation**	Painful skin **Typically, fast progression** Onset on sternum **Involvement of face** Rarely organ failure **Mucus membrane detachment**	Pruritic rash Typically, slow progression Scarlatiniform onset on the hands, feet, around ears Associated GI and, rarely, liver involvement
**Treatment**	**IVIg,** cyclosporine, systemic steroids, etanercept, infliximab, **JAK inhibitors**	**Topical steroids**, **systemic steroids**, JAK inhibitors (ex: ruxolitinib) for steroid-refractory aGVHD

Characteristics of our patient are bolded within the table.

One of the hallmark findings that differentiates aGVHD from TEN is the rapid progression and involvement of mucosal surfaces noted in classic drug-related TEN. We would expect aGVHD to clinically present with slow development of a pruritic scarlatiniform rash on the hands, feet, and ears, rarely involving the face and trunk. However, our patient presented with skin findings that rapidly progressed to a painful and blistering rash. He first developed rashes on his face, which progressed to his trunk. He had involvement of the hands and feet, but the rash was blistering, which is contrary to a typical aGVHD-related rash. He developed coalesced bullae that led to epidermal detachment with positive Nikolsky’s sign, demonstrated when lateral pressure is applied to an intact blister with resultant dislodgement of the epidermis and extension of the blister (27). In addition, our patient had significant mucus membrane involvement including his oral mucosa and genital mucosa. Our patient’s clinical presentation was not characteristic of aGVHD alone and was determined to be more consistent with TEN-like aGVHD (23, 25, 26, 28).

It is especially important to make a timely, accurate diagnosis of TEN in transplant patients, as only 20% of patients with TEN-like aGVHD reach 5-year survival. Mortality is most often due to bacteremia with sepsis due to compromised anatomical barriers to pathogens (28). Additionally, due to differences in appropriate therapeutic regimens for the conditions, it is essential to make an accurate diagnosis to provide rapid therapeutic intervention. When aGVHD is associated with a spectrum of TEN, there is a higher chance of leukopenia, diarrhea, liver dysfunction, bacteremia, hepatitis, severe dyskeratotic keratinocytes, pancytopenia, and severe thrombocytopenia than aGVHD without TEN/SJS characteristics, thus further emphasizing the importance of making an accurate diagnosis as early as possible (28). TEN does not typically respond to immunosuppression alone, prompting the addition of IVIg for our patient as described. While IVIg is an expensive intervention, it is typically well-tolerated and may provide significant benefit in this life-threatening condition, thereby potentially reducing overall expenditures accrued through escalations of care required for persistent or worsening symptoms without adequate treatment. Other treatment options for TEN-like aGVHD may include systemic steroids and cyclosporine, which our patient was already receiving. Treatment with JAK inhibition was initiated due to concern for steroid-refractory aGVHD. Targeted therapy with medications like etanercept and infliximab can be employed, although IVIg was ultimately selected for our patient as additional therapy given its more favorable side effect profile comparatively (29).

TEN-like aGVHD is an uncommon complication of stem cell transplant in general, and when it has been described in the literature, it typically has been described in adult SCT recipients or adult solid organ transplant recipients. However, cases in adult patient populations mimic our pediatric patient’s presentation. Hung et al. describe a case report of an adult status post liver transplant who presented with target lesions, folliculocentric papules, conjunctivitis, oral mucositis, renal dysfunction, and normal liver functions and was diagnosed with an SJS-like aGVHD rash that improved with the start of a TNF-alpha agent (21). A case series of two patients that presented with high fevers and a rash with mucosal involvement after a liver transplant again emphasizes the presentation of TEN-like aGVHD (24).

Goiriz et al. report a case series of 15 adult patients status post allogenic stem cell transplant who developed severe mucosal involvement and/or positive Nikolsky’s sign in the setting of blistering rash to demonstrate the difficulty in differentiating Grade 4 severe aGVHD and TEN-like aGVHD and describes a high mortality rate with this presentation (23). Finally, Macedo et al. present an adult patient status post stem cell transplant who developed severe cutaneous GVHD, ultimately diagnosed with TEN, and resolution with IVIg (22). To date, there have not been any pediatric cases reported in the literature to our knowledge.

Given the high mortality rate associated with the severity of TEN-like aGVHD and case reports from adult presentations that cited improvement from utilization of IVIg, we chose to proceed with this treatment for our pediatric patient resulting in stabilization of his rash (22, 26). We also added ruxolitinib to his treatment regimen for additional steroid-refractory aGVHD treatment due to the confounding nature of the two pathologies. Our patient is now off all immunosuppression and has no epidermal scarring or long-term sequelae of his transplant course.

The phenomenon of TEN-like aGVHD has previously only been described in adult patients and has documented poor outcomes. Due to scarce pediatric literature regarding this confounding diagnosis, identifying TEN-like aGVHD in pediatric SCT patients is a diagnostic dilemma. Our case demonstrates that a TEN spectrum should be considered as a possible epidermal reaction to allogenic SCT in the pediatric population, particularly in cases with rapid onset of blistering rash with severe mucosal involvement, and persistence despite corticosteroid therapy. Providing immediate, appropriate therapeutic intervention with IVIg, as is done for classic TEN, in SCT patients is essential in reducing morbidity and mortality, as demonstrated by our case.

### 3.1 Patient perspective

During his hospitalization, the patient and his parents report fear around the uncertainty of his diagnosis but describe clear communication between them and the medical team. The patient has recovered well post-SCT with no epidermal scarring and minimal long-term side effects from the episode described here. He has since resumed life as a typical adolescent. The patient and parents provided assent and informed consent, respectively, for this publication, which are available on request.

## Data Availability

The original contributions presented in the study are included in the article/supplementary material. Further inquiries can be directed to the corresponding author.
